# The Double-Edged Sword Effect of Entrepreneurs’ Critical Thinking on Venture Novelty

**DOI:** 10.3390/bs16061004

**Published:** 2026-06-16

**Authors:** Rui Yi, Jinzhi Luo, Yuxuan Chen, Yili Cao

**Affiliations:** 1 Business School, Xiangtan University, Xiangtan 411105, China; luojinzhi@smail.xtu.edu.cn (J.L.); chenyuxuan@smail.xtu.edu.cn (Y.C.); 2 School of Business and Management, Jilin University, Changchun 130012, China; caoyl25@mails.jlu.edu.cn

**Keywords:** critical thinking, venture novelty, interactive learning, cognitive depletion, imagination, L2, L26, D91

## Abstract

Venture novelty enables startups to overcome entry barriers and establish differentiated competitive advantages. However, research examining its antecedents from an epistemic control perspective remains limited. Drawing on survey data from 230 entrepreneurs and employing structural equation modeling (SEM) and fuzzy-set qualitative comparative analysis (fsQCA), this study investigates how entrepreneurs’ critical thinking influences venture novelty. The findings reveal a dual effect. On the one hand, critical thinking promotes venture novelty by fostering interactive learning, which facilitates the integration of heterogeneous information and the refinement of entrepreneurial opportunity insights. On the other hand, critical thinking increases cognitive depletion, thereby constraining the cognitive resources available for innovative activities. Furthermore, imagination moderates these relationships by strengthening the positive effect of interactive learning while attenuating the negative impact of cognitive depletion. FsQCA results further identify four configurational pathways to high venture novelty. This study contributes to the literature by stating both the enabling and constraining mechanisms of entrepreneurs’ critical thinking, clarifying its dual role in epistemic control, and providing configurational evidence regarding the role of imagination in fostering entrepreneurial innovation.

## 1. Introduction

In the digital era, information technologies are reshaping resource configurations and intensifying competitive dynamics across industries (Verhoef et al., 2021). Against this backdrop, startups must transcend established practices and conventional pathways to develop differentiated competitive advantages that ensure survival and sustainable growth (Rubio-Andrés et al., 2024). Venture novelty, defined as the extent to which firms introduce products, services, or business models that substantially diverge from existing offerings (Liao et al., 2024), captures both organizational originality and a forward-looking strategic posture. As a central dimension of entrepreneurial orientation, venture novelty enables firms to establish distinctive market positions, enhance organizational legitimacy, and strengthen their long-term innovative capabilities (Amason et al., 2006). However, startups typically operate under severe resource constraints and lack established routines, rendering them particularly vulnerable to information asymmetries, experiential limitations, and elevated environmental uncertainty when pursuing novel initiatives (Kaiser & Müller, 2015). These structural disadvantages complicate the process of generating and implementing novelty.

Previous studies have mostly examined entrepreneurial outcomes from the perspectives of ability and behavior (Gielnik & Bohlayer, 2024; Frese & Gielnik, 2023; Marvel et al., 2016), while paying less attention to entrepreneurs’ decision logic and action choices under limited attention. The attention-based view suggests that organizational behavior depends on how entrepreneurs allocate their limited attention to specific issues, information cues, and solutions (Ocasio, 1997). This allocation reflects entrepreneurs’ understanding and judgment of the firm’s strategic direction, core values, and priority tasks, including which problems to attend to, how many resources to allocate to solving these problems, and which problems should be addressed first (Bastian et al., 2026). Therefore, individuals need to rely on epistemic control mechanisms to complete the allocation and hierarchical ordering of attention (Mackie et al., 2013). Critical thinking is epistemic control mechanism, referring to a purposeful and self-regulated judgment process through which individuals decide “what to believe” or “what action to take” (Rauscher & Badenhorst, 2021; Bastian et al., 2026). Its formation and development may be influenced by educational experience and entrepreneurial practice, among other factors (Eggers et al., 2017; Rauscher & Badenhorst, 2021). In contexts where mature industry precedents are lacking and the information environment is increasingly complex (Amason et al., 2006), entrepreneurs face opportunity information that is ambiguous, incomplete, and uncertain. On the one hand, critical thinking helps entrepreneurs distinguish complex information, separate truth from falsehood, and grasp the core and essence of issues (Lovelace et al., 2016). On the other hand, it encourages entrepreneurs to continuously reflect on existing assumptions, break fixed thinking patterns, and optimize opportunity judgment through deep thinking (Khoshgoftar & Barkhordari-Sharifabad, 2023). Therefore, exploring how critical thinking influences venture novelty is an important issue that needs further attention from both scholars and practitioners.

Recent years, critical thinking has gradually attracted increasing attention from management scholars. Existing studies suggest that critical thinking helps individuals analyse and evaluate information, evidence, and arguments, thereby improving problem judgment (Bell & Loon, 2015; Rauscher & Badenhorst, 2021). However, some studies also indicate that critical thinking may generate cognitive load (Shehab & Nussbaum, 2015). This suggests that critical thinking has a dual nature. Yet, most existing studies have examined its positive or negative effects separately and have not fully revealed the double-edged sword effect of critical thinking within a unified theoretical framework. Combined with the attention-based view, critical thinking may influence venture novelty through different pathways. On the one hand, critical thinking directs entrepreneurs’ attention to information authenticity, evidence sufficiency, and flaws in judgment (Rauscher & Badenhorst, 2021). Since such information is difficult to obtain solely through internal experience, critical thinking encourages entrepreneurs to seek external knowledge. Interactive learning refers to the exchange and sharing of knowledge resources between entrepreneurs and stakeholders such as suppliers and customers for the purpose of promoting innovation (Meeus et al., 2001). It can help entrepreneurs integrate diverse information and broaden their cognitive boundaries (Meeus et al., 2001), thereby promoting the formation of venture novelty. On the other hand, the continuous regulation of attentional resources involved in critical thinking may lead to the overuse of attentional resources (Engin & Vetschera, 2017; Rauscher & Badenhorst, 2021), resulting in cognitive depletion. This is because entrepreneurs’ attentional resources are continuously occupied, making them more conservative in opportunity judgment and action choices (McMullen, 2015), which in turn constrains the development and implementation of venture novelty (Snihur & Zott, 2020).

Furthermore, investigating the mechanism of critical thinking requires considering the influence of contingency factors. Venture novelty is essentially a process through which entrepreneurs identify new, exploitable opportunities and thereby create new value (Amason et al., 2006). Entrepreneurs must acquire valuable, heterogeneous, and complementary knowledge resources through interactive learning to identify these new exploitable opportunities. However, when confronted with vast and complex bodies of cross-border knowledge, entrepreneurs often encounter substantial challenges in its identification and integration. Schema theory suggests that individuals rely on pre-existing cognitive schemas to interpret, structure, and respond to complex information in context-sensitive ways (Axelrod, 1973). Within this framework, imagination can be understood as a schema-based capacity for mental simulation that enables the recombination and integration of dispersed latent resources (Axelrod, 1973; Liang & Chia, 2014). On the one hand, as a process of exchanging and sharing of innovative knowledge, interactive learning relies on imagination to achieve the cross-border recombination and contextual transfer of knowledge (Liang & Chia, 2014; Meeus et al., 2001), and the interaction between the two promotes the transformation of knowledge inputs into innovation outputs. On the other hand, an efficient recombination mechanism driven by high imagination significantly conserves attention resources. Entrepreneurs can establish connections among complex resources at a lower cognitive cost (Felin & Zenger, 2009), thereby effectively weakening the impact of cognitive depletion on venture novelty. Accordingly, imagination is likely to moderate the dual pathways through which entrepreneurs’ critical thinking influences venture novelty.

This study systematically investigates how entrepreneurs’ critical thinking influences venture novelty through dual pathways, while exploring the moderating role of imagination. By doing so, it makes three primary contributions to the entrepreneurship literature. First, From the perspective of epistemic control, this study enriches research on the antecedents of venture novelty. Second, based on the attention-based view, it reveals the black box of the internal mechanism through which critical thinking influences venture novelty. Third, by integrating schema theory, it clarifies the boundary role of imagination in the effects of interactive learning and cognitive depletion on venture novelty.

## 2. Hypotheses

### 2.1. The Positive Effects of Critical Thinking: The Mediating Role of Interactive Learning

Interactive learning constitutes a central mechanism through which entrepreneurs access heterogeneous resources and enhance venture novelty (Meeus et al., 2001). Through ongoing interactions, entrepreneurs acquire industry-specific knowledge, align expectations, and mitigate resource misallocation arising from information asymmetries and cognitive inertia (Fu et al., 2013). However, for highly novel entrepreneurial activities, superficial or passive interactive learning makes it difficult to absorb and reconstruct high-value knowledge. Combined with the attention-based view, interactive learning is not simply a process of information reception, but rather a process through which entrepreneurs screen, compare, and process external knowledge under conditions of limited attention (Zahra & George, 2002; Ocasio, 1997). Therefore, whether interactive learning can be transformed into venture novelty depends on whether entrepreneurs can effectively regulate their attentional resources.

Critical thinking plays a key role in strengthening interactive learning. First, critical thinking prioritizes attention allocation, preventing attention from being occupied by non-learning goals such as emotional confrontation and position defense (Shehab & Nussbaum, 2015), thereby ensuring the stability and efficiency of interactive learning. Second, critical thinking adjusts the focus of attention allocation, shifting attention from the reception of surface-level opinions to the tracing of deeper underlying logic (Sosu, 2013), and thus promoting the transformation of interactive learning from shallow information exchange to deep cognitive co-creation. Finally, critical thinking allocates limited attention preferentially to high-value information, filters out redundant and ineffective information (Eggers et al., 2017), and improves the quality of interactive learning. In summary, we propose the following hypothesis:

**H1a.**  *Critical thinking positively influences interactive learning.*

Venture novelty depends on the support of heterogeneous knowledge (Snihur & Zott, 2020). As a core pathway through which entrepreneurs acquire external heterogeneous knowledge, interactive learning directly influences the effectiveness of venture novelty. First, sustained interactions enable entrepreneurs to cultivate trust and reciprocity with key stakeholders, thereby expanding knowledge networks and enhancing access to innovative resources (Fu et al., 2013). Second, interactive learning facilitates the identification of discrepancies between customer needs and existing market offerings, allowing entrepreneurs to detect unmet demands and uncover latent innovation opportunities (Meeus et al., 2001). Third, interactive learning promotes the active processing, interpretation, and internalization of new information, leading to the development of richer and more flexible knowledge (Johnson, 2014).

Accordingly, we propose the following hypothesis:

**H1b.**  *Interactive learning mediates the relationship between critical thinking and venture novelty. Specifically, critical thinking promotes interactive learning, which in turn enhances venture novelty.*

### 2.2. Negative Effects of Critical Thinking: The Mediating Role of Cognitive Depletion

Cognitive depletion refers to a state of mental fatigue that arises when individuals exhaust psychological and cognitive resources (Ho et al., 2022). Based on the attention-based view, entrepreneurs’ attentional resources are limited, and they can only devote time and energy to a limited number of issues (Ocasio, 1997). In the process of regulating the allocation and prioritization of attention, critical thinking itself consumes limited attentional resources (Engin & Vetschera, 2017). When the resulting cognitive load exceeds entrepreneurs’ tolerable threshold, they may actively reduce their engagement in venture novelty, fail to focus their limited attention on innovative exploration (Cheng et al., 2020) and thereby inhibit venture novelty.

Critical thinking can intensify cognitive depletion for several reasons. First, critical thinking requires entrepreneurs to prioritize attention, and continuous comparison, trade-offs, and decision-making consume limited attentional resources (Engin & Vetschera, 2017), leading to cognitive depletion. Second, critical thinking requires entrepreneurs to place the focus of their attention on complex tasks such as logical verification and creative reconstruction (Shehab & Nussbaum, 2015). This accelerates attentional consumption within a given period of time and induces cognitive depletion. Finally, critical thinking continuously adjusts the allocation of attention. Frequent attentional switching and resource reallocation increase attentional load (Engin & Vetschera, 2017), ultimately intensifying cognitive depletion.

In summary, we propose the following hypothesis:

**H2a.**  *Critical thinking positively influences cognitive depletion.*

Venture novelty is a key factor determining entrepreneurial success (Liao et al., 2024). It helps firms build differentiated barriers, avoid homogeneous competition, and enhance their market bargaining power (Amason et al., 2006). In addition, novel products, services, or business models open up new demand spaces, thereby strengthening firms’ profit potential and growth prospects (Liao et al., 2024). However, venture novelty requires entrepreneurs to possess sufficient cognitive resources to ensure information processing and attentional regulation (Amason et al., 2006; Shehab & Nussbaum, 2015). First, when individuals experience cognitive depletion, their attentional control ability is weakened (Maranges et al., 2017), making it difficult for entrepreneurs to focus on processing high-value information. Second, cognitive depletion also reduces decision-making exploration. Under cognitive depletion, entrepreneurs are more likely to adopt conservative strategies and actively avoid complex tasks that require high attentional investment (Maranges et al., 2017), thereby fundamentally inhibiting the potential for venture novelty. Finally, cognitive depletion is often accompanied by negative emotions such as anxiety and burnout, which occupy already scarce attentional resources and further inhibit entrepreneurs’ exploration and cultivation of novelty (Pekrun et al., 2002).

In summary, we propose the following hypothesis:

**H2b.**  *Cognitive depletion mediates the relationship between critical thinking and venture novelty. Specifically, critical thinking negatively affects venture novelty through cognitive depletion.*

### 2.3. The Moderating Role of Imagination

Imagination enables entrepreneurs to construct cognitively rich presentations with creative potential through information recombination across domains and envisioning alternative future states (Weick, 2006). It constitutes a critical cognitive capacity that enhances the transformation of externally acquired knowledge into novel entrepreneurial insights. Entrepreneurs with high levels of imagination are more likely to engage in iterative exchanges with stakeholders (Weick, 2006). Through scenario simulation, they systematically evaluated the feasibility, risks, and value potential of emerging opportunities (Packard et al., 2017), thereby strengthening the cognitive foundation required to assess and refine venture novelty. Moreover, imaginative entrepreneurs communicate innovative ideas to stakeholders more vividly and persuasively, mainly through gestures, visual representations, and verbal explanations (Thompson, 2018). Such effectiveness reduces stakeholders’ uncertainty, enhances relational trust, and improves access to innovation-relevant resources (Meeus et al., 2001). In contrast, entrepreneurs with lower imaginative capacity may struggle to extract novel implications from acquired knowledge or to envision alternative developmental pathways.

In summary, we proposed:

**H3a.**  *Imagination positively moderates the relationship between interactive learning and venture novelty, such that the positive effect of interactive learning on venture novelty is stronger when entrepreneurs exhibit high levels of imagination.*

From a schema-theoretic perspective, imagination reflects entrepreneurs’ capacity for mental simulation. It enables entrepreneurs to translate complex situations into manageable cognitive models via mental simulation (Cornelissen & Clarke, 2010), thereby reducing cognitive load and lowering the risk of depletion. Moreover, imagination enables entrepreneurs to transcend immediate environmental constraints and mentally prototype new products, services, or markets (Cornelissen & Clarke, 2010). As ideas become progressively elaborated through simulation and recombination, entrepreneurs maintain a future-oriented and exploratory mindset (McMullen & Kier, 2017). This forward-looking orientation buffers the motivational consequences of resource loss, reducing the context in which cognitive depletion harms innovative engagement. In contrast, entrepreneurs with lower imaginative capacity are more susceptible to rigid thinking, motivational decline, and diminished innovative effort.

In summary, we proposed the following hypothesis:

**H3b.**  *Imagination moderates the relationship between cognitive depletion and venture novelty, such that the negative effect of cognitive depletion on venture novelty is weaker when entrepreneurs exhibit high levels of imagination.*

**Figure 1:**
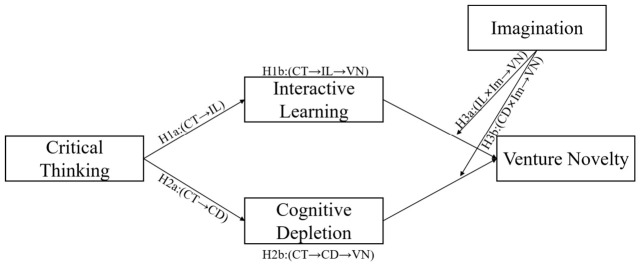
Theoretical model.

In summary, the theoretical model of this study is presented in Figure 1.

## 3. Method

### 3.1. Procedures and Samples

This study focuses on startups established within the past five years, with the objective of examining how entrepreneurs’ cognitive characteristics influence venture outcomes during the early stages of development. We employed convenience sampling and snowball sampling methods. The sample was recruited through two complementary channels. First, we collaborated with several startup incubators in Hunan and Guangdong provinces. Incubator managers facilitated access by providing contact information for eligible founders. Second, we leveraged the alumni network of the authors’ university by working with local alumni associations to invite alumni with verified entrepreneurial experience to participate in the survey. The use of multiple recruitment channels enhanced sample diversity across industries, entrepreneurial backgrounds, and venture types.

To strengthen causal inference and mitigate common method bias, we employed a two-wave survey design conducted in December 2024 (Time 1) and February 2025 (Time 2). At Time 1, we measured entrepreneurs’ critical thinking, imagination, and demographic characteristics. At Time 2, the same respondents reported their interactive learning behaviors, cognitive depletion, and venture novelty. In total, 322 valid responses were obtained at Time 1. Of the 360 entrepreneurs invited to participate in the second wave, 273 provided valid responses. To enhance sample participation, this survey also provided participants with material gifts and cash bonuses as incentives.

Rigorous data screening procedures were implemented to ensure data quality. Specifically, we: (1) remove unmatched responses across waves; (2) exclude questionnaires with uniform responses across all items; and (3) eliminate cases exhibiting clear response patterns indicative of inattentive or mechanical answering, such as straight-lining or systematic alternation. After applying these criteria, 230 fully matched and high-quality responses remained for analysis. The final sample covers a broad range of entrepreneurial sectors, including online content creation, social media marketing, food and beverage management, education and training, and financial services. This diversity enhances the external validity and practical relevance of the findings.

### 3.2. Measures

The study examined five primary constructs: critical thinking, interactive learning, cognitive depletion, imagination, and venture novelty. All measurement items were adapted from established and validated scales in prior research. Responses were recorded using a six-point Likert scale ranging from 1 (strongly disagree) to 6 (strongly agree).

Critical Thinking Disposition. Critical thinking disposition was measured using the 11-item scale developed by Sosu (2013). A sample item is: “I often re-evaluate my experiences so that I can learn from them.”, with a Cronbach’s alpha of 0.917 and composite reliability (CR) of 0.930.

Interactive Learning. Interactive learning was assessed using the 4-item scale developed by Meeus et al. (2001). A representative item is: “Firms were asked how often their suppliers contributed to their innovation processes by bringing up ideas, or participate actively.” The scale exhibited satisfactory reliability, with a Cronbach’s alpha of 0.809 and composite reliability (CR) of 0.875.

Cognitive Depletion. Cognitive depletion was assessed using the five-item scale developed by Rosen et al. (2024). A sample item is: “I had trouble concentrating.” The scale demonstrated good reliability, with a Cronbach’s alpha of 0.822 and a CR of 0.863.

Imagination. Imagination was evaluated using the nine-item scale developed by Hsu et al. (2014). A sample item is: “I like to explore unknown areas of knowledge and experience.” The scale demonstrated high reliability, with a Cronbach’s alpha of 0.892 and a CR of 0.912.

Venture Novelty. Venture novelty was assessed using the three-item scale developed by Liao et al. (2024). A sample item is: “Emily’s/Greg’s venture offers products or services that are unique to the market.” The scale demonstrated acceptable reliability, with a Cronbach’s alpha of 0.773 and a CR of 0.869.

Control Variables. Consistent with prior research on venture novelty (Liao et al., 2024), we controlled for several firm-level characteristics that may influence innovation outcomes, including firm age, number of employees, annual revenue, geographic region, and industry sector.

## 4. Data Analysis and Results

### 4.1. Common Method Bias

Harman’s single-factor test was conducted using exploratory factor analysis. The first unrotated principal component accounted for 33.44% of the total variance, which is below the commonly accepted threshold of 40%. This result suggests that no single factor explains the majority of covariance among the measures in the present study. Second, variance inflation factors (VIF) were calculated to assess multicollinearity and potential common method inflation. All VIF values were below 3, indicating that multicollinearity is not a serious concern and further reducing the likelihood that common method bias significantly distorts the observed relationships.

### 4.2. Reliability and Validity

All constructs in this study demonstrated satisfactory internal consistency. Cronbach’s alpha and CR values for all variables exceeded the recommended threshold of 0.70. The Kaiser-Meyer-Olkin (KMO) measure was 0.916, well above the recommended minimum of 0.50, and Bartlett’s test of sphericity was significant (*p* < 0.001), supporting the adequacy of the data structure.

CFA was conducted to evaluate the measurement model, with the results summarized in Table 1. The hypothesized five-factor model exhibited a good fit to the data (χ^2^ = 791.792, df = 454, χ^2^/df = 1.744; RMSEA = 0.057; SRMR = 0.0596; CFI = 0.905; TLI = 0.896). All fit indices met or closely approached recommended thresholds, indicating acceptable overall model fit. These results support both the construct validity and discriminant validity of the measures. Furthermore, the average variance extracted (AVE) for all constructs exceeded 0.50. As shown in Table 2, the square roots of AVE (reported on the diagonal) were greater than the corresponding inter-construct correlations, indicating adequate discriminant validity among constructs.

### 4.3. Hypothesis Test

**Figure 2:**
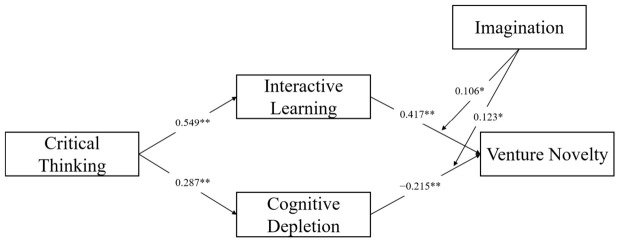
Statistical result of the model. Notes: *n* = 230; * *p* < 0.05; ** *p* < 0.01.

The structural equation model was estimated using SmartPLS 4.0 (Figure 2). Results indicated that critical thinking has a significant positive effect on interactive learning (β = 0.549, *p* < 0.001) and cognitive depletion (β = 0.287, *p* < 0.001). These findings support Hypotheses H1a and H2a. Bootstrapped procedures were used to test the proposed mediating mechanisms. Interactive learning significantly mediated the relationship between critical thinking and venture novelty (β = 0.229, 95% CI [0.124, 0.346], *p* < 0.001). Cognitive depletion also exerted a significant mediating effect (β = −0.062, 95% CI [−0.108, −0.024], *p* < 0.05). These results provide empirical support for Hypotheses H1b and H2b.

**Figure 3:**
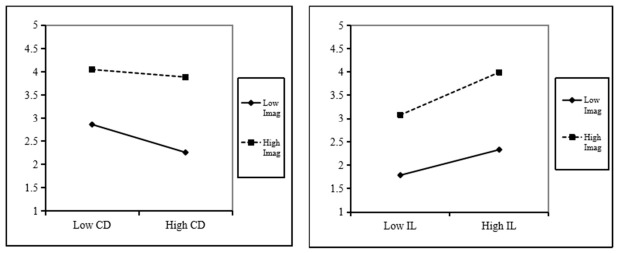
Moderating role of leader competence. Notes: CD: Cognitive depletion; IL: Interactive learning; Imag: Imagination.

To test the moderating role of imagination, interaction terms were constructed using the product indicator approach. Results show that the interaction between imagination and interactive learning is positively associated with venture novelty (β = 0.106, *p* < 0.05). This indicates that imagination strengthens the positive effect of interactive learning on venture novelty. Simple slope analysis further reveals that under high imagination (Figure 3), the effect of interactive learning on venture novelty is stronger (β = 0.523, *p* < 0.01). Under low imagination, the effect remains positive but weaker (β = 0.311, *p* < 0.01). Thus, H3a is supported. Similarly, the interaction between imagination and cognitive depletion is significant and positive (β = 0.123, *p* < 0.05), suggesting that imagination buffers the negative impact of cognitive depletion. Simple slope analysis indicates that the negative effect of cognitive depletion on venture novelty was stronger under low imagination (β = −0.338, *p* < 0.01) but nonsignificant under high imagination (β = −0.091, *p* > 0.05), supporting Hypothesis H3b.

### 4.4. fsQCA Analysis

Building on the structural equation modeling results, we employed fuzzy-set qualitative comparative analysis (fsQCA) to explore the configurational antecedents of venture novelty. Unlike symmetric net-effect approaches such as SEM, fsQCA allows for the examination of causal complexity, equifinality, and asymmetric relationships among antecedent conditions. This method is particularly suitable for uncovering multiple pathways through which combinations of critical thinking, interactive learning, cognitive depletion, and imagination jointly contribute to high venture novelty.

Variable Calibration. All variables were calibrated into fuzzy sets using the direct calibration method. The three specified qualitative anchors were the full membership at 0.95, the crossover point at 0.50, and the full non-membership at 0.05. Consistent with Fiss (2011), cases with membership scores exactly at the crossover point (0.50) were adjusted by subtracting 0.001 to avoid ambiguity in set assignment fsQCA.

Single-Condition Necessity Analysis. A necessity analysis was conducted to determine whether any single antecedent condition is required for achieving high venture novelty. As shown in Table 3, the consistency values for all antecedent conditions were below 0.90, indicating that no single condition constitutes a necessary prerequisite for venture novelty.

Configurational Sufficiency Analysis. We then conducted a sufficiency analysis to identify combinations of antecedent conditions associated with high venture novelty. Critical thinking, interactive learning, cognitive depletion, and imagination were specified as causal conditions, as these constructs are theoretically grounded in the entrepreneurship and cognitive resource literature. Following established fsQCA guidelines, the minimum consistency threshold for sufficiency was set at 0.80. Additionally, the raw coverage to proportional reduction in inconsistency (RPI) threshold was set at 0.60 (Muraven & Baumeister, 2000). Configurational analyses were conducted using both the intermediate and parsimonious solutions. Following the procedure outlined by Fiss (2011), Table 4 presents four configurations leading to high venture novelty. The overall solution consistency is 0.827, exceeding the recommended threshold of 0.837 (>0.50), indicating that the model demonstrates strong empirical relevance and explanatory power.

A1: Learning-driven resource preservation mode

The configuration with interactive learning and the absence of cognitive depletion as core conditions can promote a high level of venture novelty. This is because the continuous absorption and repeated integration of external heterogeneous knowledge enable entrepreneurs to constantly revise their innovation direction and enhance the richness and breakthrough potential of their ideas. Meanwhile, a low level of cognitive depletion ensures that they have sufficient attentional and processing resources to complete these cognitive processes. Tang et al. (2025) argue that internal and external connections can, on the one hand, provide information and resources and enhance creativity, but on the other hand, they may also consume individual resources and trigger ego depletion, thereby hindering creative performance. Therefore, when entrepreneurs are in a state of low cognitive depletion, their limited attentional resources can be continuously devoted to the identification, screening, and integration of external information, allowing interactive learning to be effectively transformed into a high level of venture novelty.

A2: Learning–imagination recombination mode

The configuration with interactive learning and imagination as core conditions can promote a high level of venture novelty. Interactive learning provides entrepreneurs with market information, technological knowledge, and resource cues from external actors such as customers, suppliers, and partners, while imagination helps entrepreneurs transform this external knowledge into future-oriented ideas for new products, new services, or new business models. Kier and McMullen (2018) noted that entrepreneurial imagination is not free fantasy detached from real-world knowledge, but rather the ability to generate and select new ideas through mental simulation by integrating task-relevant knowledge. The results of this configuration indicate that interactive learning and entrepreneurial imagination need to work synergistically. Venture novelty does not simply depend on the input of external knowledge, nor does it arise solely from individual imagination. Instead, it is gradually formed through the combination of the two and the reconstruction of knowledge.

A3: Analytical–imaginative balanced mode

The configuration with critical thinking, imagination, and the absence of cognitive depletion as core conditions can promote a high level of venture novelty. The formation of venture novelty requires imagination to break through existing experience and real-world constraints, thereby generating ideas for new products, new services, or new business models. At the same time, it also requires critical thinking to analyse, compare, and judge the feasibility of these ideas. When cognitive depletion is low, these two cognitive resources can function smoothly and reinforce each other, forming a dual chain of “logical analysis–creative extension”, thereby significantly enhancing the level of innovation. Rodet (2022) pointed out that creativity does not rely solely on free association or idea generation, but involves both idea generation and idea evaluation. The generation process helps produce novel possibilities, while the evaluation process helps screen, revise, and refine creative ideas. Therefore, the results of this configuration indicate that only when entrepreneurs are in a state of low cognitive depletion can they simultaneously maintain creative imagination and rational evaluation, thereby promoting the formation of a high level of venture novelty.

A4: Imagination-compensatory mode under cognitive strain

The configuration with the absence of critical thinking, cognitive depletion, and imagination as core conditions can promote a high level of venture novelty. When entrepreneurs lack critical thinking and experience a high level of cognitive depletion, a high level of imagination can still sustain innovative output. Zhu et al. (2017) showed that creative judgment does not rely entirely on deep thinking; rather, intuitive processing may be more helpful in selecting highly original ideas. This pathway supports this view. When entrepreneurs find it difficult to advance opportunity judgment through systematic analysis, evidence evaluation, and continuous revision, a high level of imagination may still connect fragmented information in an intuitive, scenario-based, and future-oriented manner, thereby forming novel ideas for products, services, or business models. Therefore, this configuration does not deny the value of critical thinking. Instead, it demonstrates that, under conditions of constrained cognitive resources, venture novelty can be achieved through the pathway of imagination.

## 5. Discussion

This study draws on Attention-Based View and Schema Theory and investigates how entrepreneurs’ critical thinking influences venture novelty. We uncover a nuanced and dual-path “double-edged” underlying entrepreneurial innovation. Moreover, The results demonstrate that imagination strengthens the positive effect of interactive learning on venture novelty while mitigating the negative effect of cognitive depletion. Finally, the configurational analysis deepens these insights by identifying four distinct pathways leading to high venture novelty.

### 5.1. Theoretical Contributions

First, this study extends research on venture novelty from epistemic control perspective. Drawing on the attention-based view, this study conceptualizes critical thinking as a cognitive regulatory mechanism that influences the allocation of cognitive resources, and reveals its double-edged sword effect on venture novelty through interactive learning and cognitive depletion. Specifically, on the one hand, critical thinking helps entrepreneurs allocate attention to information with greater exploratory value. Through interactive learning, entrepreneurs can compare, verify, and supplement such information, thereby promoting venture novelty. On the other hand, critical thinking may also occupy limited attentional resources because of sustained cognitive investment, leading to cognitive depletion and inhibiting venture novelty. Thus, this study not only responds to Grégoire et al. (2011) call for entrepreneurial cognition research to shift toward the dynamic interaction between cognitive resources and mental representations, but also responds to Bastian et al. (2026) identified research gap regarding how epistemic control mobilizes attention resources under uncertainty and influences venture novelty.

Second, this study reveals the double-edged sword effect of critical thinking on venture novelty. Previous studies have mainly emphasised the positive role of critical thinking (Bell & Loon, 2015; Rauscher & Badenhorst, 2021; Thornhill-Miller et al., 2023). However, some studies have also suggested that critical thinking may generate cognitive load and thereby inhibit innovation (Shehab & Nussbaum, 2015). Therefore, this study introduces interactive learning and cognitive depletion as two mediators to explain why critical thinking may exert differentiated effects on venture novelty. In doing so, this study responds to discussions in entrepreneurship research that specific cognitive or motivational antecedents may shift from positive to negative effects during the entrepreneurial process (Gielnik et al., 2018; Hashai & Zahra, 2022).

Third, based on schema theory, this study reveals the boundary role of imagination in the formation of venture novelty. Prior research has predominantly conceptualized imagination as a stable dispositional trait (Thompson, 2018), while paying less attention to how it influences entrepreneurs’ integration and recombination of complex knowledge. This study conceptualizes imagination as a mental simulation ability based on cognitive schemas, arguing that it helps entrepreneurs establish new connections among dispersed, heterogeneous, and cross-domain knowledge. Thus, imagination not only contributes to the formation of novel ideas, but also changes the effects of interactive learning and cognitive depletion on venture novelty. Therefore, this study reveals how imagination influences the formation of venture novelty under different cognitive and learning conditions, thereby responding to discussions in entrepreneurial imagination research on how imagination promotes opportunity imagining, opportunity rationalization, and new idea generation (Cornelissen & Clarke, 2010; Kier & McMullen, 2018).

Finally, the fsQCA results extend the theoretical model of this study by revealing the configurational and asymmetric nature of entrepreneurial cognition. The configurational results show that high venture novelty can be achieved through multiple cognitive patterns, including learning-driven resource preservation, learning–imagination integration, analytical–imaginative balance, and imagination-based compensation under cognitive strain. This indicates that the role of critical thinking is neither linear nor universally beneficial, but depends on how it is combined with behavior and imagination. In particular, the imagination-compensatory configuration reflects clear causal asymmetry: the absence of critical thinking and the presence of cognitive depletion do not necessarily hinder high venture novelty. This finding deepens entrepreneurial cognition research by showing that different cognitive resources may substitute for, complement, or compensate for one another in the formation of venture novelty.

### 5.2. Practical Implications

First, entrepreneurs should intentionally cultivate critical thinking to strengthen their firms’ innovation capabilities. The formation and development of critical thinking may be influenced by educational experience, entrepreneurial practice, and sociocultural contexts. Therefore, entrepreneurs can continuously improve their critical thinking through reflective training, entrepreneurial review, and cross-domain communication. However, critical thinking may also increase cognitive load and delay timely decision-making. Thus, entrepreneurs should use critical thinking prudently to avoid its potential adverse effects.

Second, firms should institutionalize structured scenario simulations and creative brainstorming sessions to provide entrepreneurs with a psychologically safe environment for experimentation and opportunity exploration. Entrepreneurs can subsequently validate these ideas through small-scale pilot initiatives and rapid feedback mechanisms, enabling iterative refinement and facilitating the effective transformation of imaginative insights into implementable innovations.

Finally, organizations and institutions should cultivate a work environment that promotes continuous learning and supports cognitive recovery to sustain long-term innovation performance. The findings suggest that even when entrepreneurs exhibit relatively lower levels of critical thinking or imagination, they may still achieve venture novelty by engaging in high-quality interactive learning while effectively managing cognitive load. By fostering open communication networks and implementing structured recovery practices such as periodic breaks and team support, entrepreneurs can maintain manageable cognitive demands and preserve the cognitive resources necessary for sustained innovation.

### 5.3. Limitations and Directions of Future Research

Although this study provides novel theoretical insights, it shows several limitations. First, the research is based on a cross-sectional survey design. Despite efforts to mitigate common method bias through multi-wave, multi-source data collection and the application of multiple statistical techniques, the dynamic relationships and causal linkages among the focal variables cannot be definitively established. Future research could employ quasi-experimental designs, longitudinal panel data, or experience-sampling methods to better capture temporal dynamics and strengthen causal inference.

Second, this study focuses only on the antecedent formation mechanism of venture novelty, without further exploring the outcomes generated by venture novelty. Future research could examine entrepreneurial outcome variables such as entrepreneurial performance and entrepreneurial resilience, and conduct further studies on the subsequent effects of venture novelty. Given that venture novelty is a factor influencing entrepreneurial success, future research could further extend the research chain and investigate in depth the mechanisms and boundary conditions through which venture novelty affects entrepreneurial success.

Third, this study explains the internal mechanism through which critical thinking influences venture novelty via mediators from epistemic control perspective. However, in practice, entrepreneurs are constrained by objective factors such as the external market environment and the current conditions of the firm. Even when entrepreneurs possess critical thinking, they may still choose a more conservative and less novel entrepreneurial path based on practical considerations. Future research could further incorporate external contextual factors and firm-level characteristics to explore the differentiated effects of entrepreneurial thinking on venture novelty.

Finally, the variables in this study were measured through entrepreneurs’ self-reports, which may be influenced by subjective judgment and temporary situations. For example, cognitive depletion may be affected by measurement timing, recent workload, short-term emotions, or unexpected events. Therefore, the cognitive depletion measured in this study is closer to a perceived state during the survey period, rather than a completely stable psychological state. Future research could adopt experience sampling methods, diary studies, or repeated measurements at multiple time points to better capture its dynamic changes. In addition, venture novelty was also based on entrepreneurs’ self-evaluations. Entrepreneurs may overestimate or underestimate the uniqueness of their ventures due to self-confidence, impression management, or insufficient market feedback. Future research could combine external indicators such as customer evaluations, patent records, and product launch data to further verify the consistency between perceived novelty and actual market novelty.

## Figures and Tables

**Table 1:** Confirmatory factor analysis.

Model	Factor Structure	χ^2^	df	χ^2^/df	RMSEA	SRMR	CFI	IFI
Five-factor model	CT, IL, CD, Imag, VN	791.792	454	1.744	0.057	0.0596	0.905	0.906
Four-factor model	CT, IL + CD, Imag, VN	1232.208	458	2.690	0.086	0.1636	0.782	0.785
Three-factor model	CT, IL + CD + Imag, VN	1283.544	461	2.784	0.088	0.0962	0.769	0.771
Dual-factor model	CT, IL + CD + Imag + VN	1398.089	463	3.02	0.094	0.0998	0.737	0.740
Single-factor model	CT + IL + CD + Imag + EN	1767.361	464	3.809	0.111	0.1093	0.634	0.637

Note: CT = Critical Thinking; IL = Interactive Learning; CD = Cognitive Depletion; VN = Venture Novelty; Imag = Imagination.

**Table 2:** Descriptive statistics and correlation coefficients.

Variables	Mean	SD	CT	IL	CD	VN	Imag	FA	NOE	AR	Region	Id
CT	4.457	0.835	(0.74)									
IL	4.313	0.873	0.545 **	(0.797)								
CD	3.435	0.884	0.233 **	0.032	(0.749)							
VN	4.035	0.943	0.280 **	0.504 **	−0.122	(0.83)						
Imag	4.255	0.804	0.620 **	0.640 **	0.060	0.454 **	(0.732)					
FA	3.1	0.96	0.030	0.060	−0.075	0.009	−0.005	--				
NOE	2.49	0.875	0.087	0.014	0.059	0.136 *	0.022	0.122	--			
AR	2.68	1.003	0.092	0.033	0.083	0.229 **	0.124	0.234 **	0.554 **	--		
Region	2.14	0.992	−0.201 **	−0.229 **	0.081	−0.063	−0.123	−0.154 *	0.117	0.093	--	
Id	3.05	1.483	−0.012	0.031	0.150 *	−0.010	−0.004	−0.185 **	−0.016	−0.036	0.081	--

Notes: *n* = 230; FA = firm age; NOE = number of employees; AR = annual revenue; Id = industry; *: *p* < 0.05, **: *p* < 0.01; and the diagonal entries display the square roots of the AVE.

**Table 3:** Necessity analysis results.

Variables	Venture Novelty	
	Consistency	Coverage
CT	0.768901	0.730126
~CT	0.555789	0.581235
IL	0.811743	0.786559
~IL	0.540081	0.55262
CD	0.645641	0.619713
~CD	0.69266	0.681819
Imag	0.804964	0.788312
~Imag	0.539994	0.546439

Note: CT = Critical Thinking; IL = Interactive Learning; CD = Cognitive Depletion; VN = Venture Novelty; Imag = Imagination.

**Table 4:** Configuration analysis results.

Conditional Variable	A1	A2	A3	A4
CT			●	◎
IL	●	●		
CD	◎		◎	●
Imag		●	●	●
Consistency	0.86392	0.85149	0.876545	0.895808
Coverage	0.590236	0.724599	0.50739	0.366091
Unique coverage	0.0634862	0.11067	0.0164326	0.0181363
Coverage of solutions	0.836982			
Consistency of solutions	0.826798			

Note: CT = Critical Thinking; IL = Interactive Learning; CD = Cognitive Depletion; VN = Venture Novelty; Imag = Imagination. “●” refers to the existence of core conditions; “◎” denotes the missing of core conditions.

## Data Availability

The data used to support the findings of this study are available from the corresponding author upon request.

## Abbreviations

The following abbreviations are used in this manuscript:

SEM	Structural equation modeling
KMO	Kaiser-Meyer-Olkin
fsQCA	Fuzzy-set qualitative comparative analysis
COR	Conservation of resources
VIF	Variance inflation factors
